# Natural Antioxidants in Obesity and Related Diseases

**DOI:** 10.3390/antiox12111966

**Published:** 2023-11-05

**Authors:** Hyo In Kim, Jinbong Park

**Affiliations:** 1 Department of Surgery, Beth Israel Deaconess Medical Center, Harvard Medical School, Boston, MA 02215, USA; hkim21@bidmc.harvard.edu; 2 Department of Pharmacology, College of Korean Medicine, Kyung Hee University, Seoul 02447, Republic of Korea

Obesity is a chronic complex disease defined by excessive adiposity that impairs health. Obesity not only increases the risk of metabolic diseases such as diabetes, cardiovascular diseases, and fatty liver diseases but also produces a chronic inflammatory state which leads to many complications [1]. Recent studies have demonstrated that increased fat deposition in obesity may play a role in or be a result of excessive oxidative stress in the body. Although recent medications for obesity show promising results, further evidence is still required [2]. Antioxidants modulate oxidative stress, and natural antioxidants are widely distributed in food and medicinal plants. These natural antioxidants exhibit a wide range of biological effects, including anti-inflammatory [3], anti-aging [4], anti-obesity [5,6] and anti-cancer [7]. The consumption of natural antioxidants can ameliorate damage caused by oxidative stress through inhibiting the oxidative chain reaction by acting as free radical scavengers. Understanding of the mechanism of action of natural antioxidants in redox modulation could be useful to prevent or develop therapies for obesity and related diseases such as diabetes, cardiovascular diseases, fatty liver diseases, inflammatory diseases or even cancer. Thus, our Special Issue “Natural Antioxidants in Obesity and Related Diseases” was assembled to contribute to the search for these natural antioxidants and provide advances in therapeutic interventions for obesity and related diseases. This Special Issue contains thirteen contributions, five reviews and eight research articles. The list of contributions is as below:

1. Souza, F.; Silva, G.; Cadavid, C.; Lisboa, L.; Silva, M.; Paiva, W.; Ferreira, M.; de Paula Oliveira, R.; Rocha, H. Antioxidant *Baccharis trimera* Leaf Extract Suppresses Lipid Accumulation in C. elegans Dependent on Transcription Factor NHR-49. Antioxidants 2022, 11(10), 1913; https://doi.org/10.3390/antiox11101913.
2. Park, W.; Song, G.; Boo, M.; Kim, H.; Park, J.; Jung, S.; Choi, M.; Kim, B.; Kim, Y.; Kim, M.; Kim, K.; Kwak, H.; Leem, J.; Um, J.; Park, J. Anmyungambi Decoction Ameliorates Obesity through Activation of Non-Shivering Thermogenesis in Brown and White Adipose Tissues. Antioxidants 2023, 12(1), 49; https://doi.org/10.3390/antiox12010049.
3. Tamel Selvan, K.; Goon, J.; Makpol, S.; Tan, J. Effects of Microalgae on Metabolic Syndrome. Antioxidants 2023, 12(2), 449; https://doi.org/10.3390/antiox12020449.
4. Arellano-García, L.; Trepiana, J.; Martínez, J.; Portillo, M.; Milton-Laskibar, I. Beneficial Effects of Viable and Heat-Inactivated *Lactobacillus rhamnosus* GG Administration on Oxidative Stress and Inflammation in Diet-Induced NAFLD in Rats. Antioxidants 2023, 12(3), 717; https://doi.org/10.3390/antiox12030717.
5. Méndez, L.; Muñoz, S.; Barros, L.; Miralles-Pérez, B.; Romeu, M.; Ramos-Romero, S.; Torres, J.; Medina, I. Combined Intake of Fish Oil and D-Fagomine Prevents High-Fat High-Sucrose Diet-Induced Prediabetes by Modulating Lipotoxicity and Protein Carbonylation in the Kidney. Antioxidants 2023, 12(3), 751; https://doi.org/10.3390/antiox12030751.
6. Zheng, Y.; Lee, S.; Lee, Y.; Lee, T.; Kim, J.; Kim, T.; Kang, I. Standardized Sanguisorba officinalis L. Extract Inhibits Adipogenesis and Promotes Thermogenesis via Reducing Oxidative Stress. Antioxidants 2023, 12(4), 882; https://doi.org/10.3390/antiox12040882.
7. Munteanu, C.; Schwartz, B. The Effect of Bioactive Aliment Compounds and Micronutrients on Non-Alcoholic Fatty Liver Disease. Antioxidants 2023, 12(4), 903; https://doi.org/10.3390/antiox12040903.
8. Jung, U. Sarcopenic Obesity: Involvement of Oxidative Stress and Beneficial Role of Antioxidant Flavonoids. Antioxidants 2023, 12(5), 1063; https://doi.org/10.3390/antiox12051063.
9. Munkhsaikhan, U.; Kwon, Y.; Sahyoun, A.; Galán, M.; Gonzalez, A.; Ait-Aissa, K.; Abidi, A.; Kassan, A.; Kassan, M. The Beneficial Effect of Lomitapide on the Cardiovascular System in LDLr^-/-^ Mice with Obesity. Antioxidants 2023, 12(6), 1287; https://doi.org/10.3390/antiox12061287.
10. Naomi, R.; Teoh, S.; Halim, S.; Embong, H.; Hasain, Z.; Bahari, H.; Kumar, J. Unraveling Obesity: Transgenerational Inheritance, Treatment Side Effects, Flavonoids, Mechanisms, Microbiota, Redox Balance, and Bioavailability & mdash; A Narrative Review. Antioxidants 2023, 12(8), 1549; https://doi.org/10.3390/antiox12081549.
11. Mokgalaboni, K.; Dlamini, S.; Phoswa, W.; Modjadji, P.; Lebelo, S. The Impact of Punica granatum Linn and Its Derivatives on Oxidative Stress, Inflammation, and Endothelial Function in Diabetes Mellitus: Evidence from Preclinical and Clinical Studies. Antioxidants 2023, 12(8), 1566; https://doi.org/10.3390/antiox12081566.
12. Cortés-Espinar, A.; Ibarz-Blanch, N.; Soliz-Rueda, J.; Bonafos, B.; Feillet-Coudray, C.; Casas, F.; Bravo, F.; Calvo, E.; Ávila-Román, J.; Mulero, M. Rhythm and ROS: Hepatic Chronotherapeutic Features of Grape Seed Proanthocyanidin Extract Treatment in Cafeteria Diet-Fed Rats. Antioxidants 2023, 12(8), 1606; https://doi.org/10.3390/antiox12081606.
13. Dave, A.; Park, E.; Pezzuto, J. Multi-Organ Nutrigenomic Effects of Dietary Grapes in a Mouse Model. Antioxidants 2023, 12(10), 1821; https://doi.org/10.3390/antiox12101821.

The first contribution to this Special Issue was an original research article entitled “Antioxidant Baccharis trimera Leaf Extract Suppresses Lipid Accumulation in C. elegans Dependent on Transcription Factor NHR-49” by Souza et al. (Contribution 1). The authors investigated the effect of *Baccharis tirmera* leaf extract (BT) on lipid accumulation. BT is a medicinal plant used to treat obesity in Brazilian folk medicine, rich in phenolic compounds including rutin, hyperoside and 5-caffeoylquinic acid. Through a series of experiments using 3T3 murine fibroblasts or *Caenorhabditis elegans*, the authors clearly showed the antioxidant activity of BT, which was an effect independent of the presence of transcription factors related to stress resistance (SKN-1 and DAF-16). Rather, NHR-49, a transcription factor homologous to the mammalian hepatocyte nuclear factor 4 alpha (HNF4α), and which regulates the β-oxidation and fatty acid desaturation pathways, was critical for the antioxidant activity of BT.

Next, our group, as a team of the College of Korean Medicine of Kyung Hee University, studied a clinical-based multiherbal decoction named Anmyungambi (AMGB) decoction. In “Anmyungambi Decoction Ameliorates Obesity through Activation of Non-Shivering Thermogenesis in Brown and White Adipose Tissues” (Contribution 2), we show that AMGB decoction alleviates high-fat diet (HFD)-induced obesity in C57BL/6 mice. In particular, we focus on its effect on non-shivering thermogenesis, a recently discovered novel pathway of energy expenditure. Although the clinical evidence [8] and potential mechanisms [9] of AMGB decoction has been accumulated through previous studies, this is the first study to demonstrate how this herbal medicine can decrease lipid accumulation induced by HFD.

Microalgae, unicellular and photosynthetic microscopic algae that live in fresh or saltwater, are emerging sources of bioactive components such as polysaccharides, carotenoids and pigments. These bioactive compounds exert antioxidant, anti-inflammatory, anti-obesity, antihypertensive, hepatoprotective and immunomodulatory effects, so the potential of microalgae as health supplements is gaining interest [10]. The review article by Tamel Selvan, “Effects of Microalgae on Metabolic Syndrome” (Contribution 3), outlines the possible use of microalgae as health supplements for metabolic syndromes including abdominal obesity, hypertension, hypertriglyceridemia, reduced high-density lipoprotein cholesterol and hyperglycemia.

The gastrointestinal tract is a key factor in non-alcoholic fatty liver disease (NAFLD) pathophysiology. In particular, the gut microbiota and their balance are critical. In “Beneficial Effects of Viable and Heat-Inactivated *Lactobacillus rhamnosus* GG Administration on Oxidative Stress and Inflammation in Diet-Induced NAFLD in Rats” by Arellano-García et al. (Contribution 4), the authors demonstrate that the viable probiotic *Lactobacillus rhamnosus* GG and its heat-inactivated parabiotic form exert antioxidant properties in a high-fat high-fructose (HFHF)-induced NAFLD rat model. Both live and heat-inactivated *Lactobacillus rhamnosus* GG prevented the oxidative stress and the inflammation induced in the liver by HFHF. Interestingly, the parabiotic was even more effective than the originating probiotic. This may be a great advantage, since a viable microbiome is also a source which can possibly induce systemic infection and subsequent immune stimulation. The authors expect that the potential role of lysozyme contributes to the greater antioxidant effect of the heat-inactivated parabiotic *Lactobacillus rhamnosus* GG.

Insulin resistance with oxidative stress and inflammation can lead to kidney diseases. It is considered an early metabolic alteration in chronic kidney disease (CKD) patients and is related to increased risk for CKD in nondiabetic patients [11]. Méndez et al., in “Combined Intake of Fish Oil and D-Fagomine Prevents High-Fat High-Sucrose Diet-Induced Prediabetes by Modulating Lipotoxicity and Protein Carbonylation in the Kidney” (Contribution 5), show that fish oil and D-fagomine can alleviate CKD in high-fat high-sucrose (HFHS)-fed rats. Fish oil has beneficial health effects mainly due to its high content of ω-3 fatty acids such as eicosapentaenoic (EPA) and docosahexaenoic (DHA) acids [12]. D-Fagomine is a bioactive component mainly found in buckwheat-based food products [13], known for its positive effect on glucose tolerance [14]. The authors demonstrate that the combination of fish oil with D-Fagomine may have suppressive effects on the progression of renal dysfunction in a prediabetic state through amelioration of fat accumulation and decreased oxidative damage in the kidney.

*Sanguisorba officinalis* L. (SO) is a medicinal herb widely distributed in East Asia. Studies demonstrate that SO has antioxidant properties [15]. In “Standardized Sanguisorba officinalis L. Extract Inhibits Adipogenesis and Promotes Thermogenesis via Reducing Oxidative Stress” by Zheng et al. (Contribution 6), the authors demonstrate that standardized SO shows an anti-obesity effect in 3T3-L1 adipocytes and HFD-induced obese mice through antioxidant pathways. SO at 200 mg/kg or above reduced ROS production and oxidative stress. AMP-activated protein kinase, peroxisome proliferator activated receptor alpha, uncoupling protein 1 and arnitine palmitoyltransferase 1 were involved in the effect of SO.

NAFLD is a chronic liver disease defined as an excessive accumulation of fat, mainly in the form of triglycerides in the liver epithelial cells or hepatocytes. The incidence rate of NAFLD is around 30%, and its prevalence increases significantly between 70 and 90% among obesity or type 2 diabetes (T2D) patients [16]. The disease progression of NAFLD is based on a complex network of factors including insulin resistance, oxidative stress, genetic and epigenetic mechanisms, environmental elements, cytokines and changes in the microbiota [17]. Munteanu and Schwartz, the authors of the article “The Effect of Bioactive Aliment Compounds and Micronutrients on Non-Alcoholic Fatty Liver Disease” (Contribution 7), reviewed studies on food-based compounds or micronutrients that may interfere with NAFLD, such as dark chocolate, cocoa butter, peanut butter, sweeteners, glutathione, soy lecithin, silymarin, Aquamin, cannabinoids and vitamins.

While obesity epidemics are rising, aging is another important health issue worldwide. Sarcopenic obesity, characterized by decreased muscle mass, strength and performance along with abnormally excessive fat mass, is an age-related disease which can lead to further comorbidities such as T2D, NAFLD, dyslipidemia and cardiovascular diseases [18]. The review article “Sarcopenic Obesity: Involvement of Oxidative Stress and Beneficial Role of Antioxidant Flavonoids” by Jung (Contribution 8) describes the general characteristics and pathophysiology of sarcopenic obesity, particularly focusing on the role of oxidative stress within the possible mechanisms. The author also discusses the potential benefits of antioxidant flavonoids to treat sarcopenic obesity.

Lomitapide is an inhibitor of microsome triglyceride transfer protein (MTP) approved by the Food and Drug Administration (FDA). Lomitapide directly binds to MTP and prevents lipid transfer [19]. Munkhsaikhan et al. investigate whether lomitapide shows beneficial effects on the cardiovascular system using LDL receptor knockout (LDLr^−/−^) mice to demonstrate its mechanism of action in homozygous familial hypercholesteremia (HoFH) in a research article entitled “The Beneficial Effect of Lomitapide on the Cardiovascular System in LDLr^−/−^ Mice with Obesity” (Contribution 9). HoFH is a rare but critical metabolic disease derived from LDL receptor mutation [20]. The authors show that body weight, fat mass, blood glucose, lipid levels and atherosclerotic plaque area were decreased by lomitapide treatment in HFD-fed LDLr^−/−^ mice. Also, lean mass and endothelium function in the thoracic aorta and mesenteric resistance artery were improved. This effect of lomitapide was due to decreased endoplasmic reticulum stress, oxidative stress and inflammation.

A review article by Naomi et al., entitled “Unraveling Obesity: Transgenerational Inheritance, Treatment Side Effects, Flavonoids, Mechanisms, Microbiota, Redox Balance, and Bioavailability—A Narrative Review” (Contribution 10), also describes the beneficial role of flavonoids. Flavonoids can reduce adipose tissue mass and the formation of intracellular free radicals, enhance endogenous antioxidant defenses, modulate the redox balance and reduce inflammatory signaling pathways. The authors review 31 articles published in Wiley Online Library, Scopus, Nature and ScienceDirect during the past decade. This review provides an insight into the domain of a natural product therapeutic approach for managing obesity and recapitulates the transgenerational inheritance of obesity, the current available treatments to manage obesity and its side effects, flavonoids and their sources, the molecular mechanism involved, the modulation of gut microbiota in obesity, redox balance, and the bioavailability of flavonoids.

Another review article published in this Special Issue focuses on a certain plant. Mokgalaboni et al. outline the scientific evidence of the anti-oxidant, anti-inflammatory and endothelial functions of *Punica granatum* Linn, more well known as pomegranate, in “The Impact of *Punica granatum* Linn and Its Derivatives on Oxidative Stress, Inflammation, and Endothelial Function in Diabetes Mellitus: Evidence from Preclinical and Clinical Studies” (Contribution 11). From 170 studies in PubMed, Scopus and Google Scholar, the authors reviewed 46 papers (33 preclinical studies and 13 clinical studies) on the effect of pomegranate on T2D. Pomegranate shows promising results as a potential agent for improving oxidative stress, inflammation and endothelial dysfunction in diabetes. Still, the authors mention that further clinical trials with a powered sample size is necessary to fully determine its beneficial effect.

Cortés-Espinar et al., in “Rhythm and ROS: Hepatic Chronotherapeutic Features of Grape Seed Proanthocyanidin Extract Treatment in Cafeteria Diet-Fed Rats” (Contribution 12), used cafeteria diet (CAF)-fed Fischer 344 rats to study the effect of grape seed proanthocyanidin extract (GSPE) on metabolic diseases. CAF consists of biscuits with cheese and pâté, bacon, ensaimada (pastry), standard chow, carrots and milk containing 22% sucrose. The CAF induced hepatic antioxidant effects (AOX), and GSPE alleviated this. Since AOX and the circadian system are closely connected [21], the authors further studied the effect of GSPE on the liver-central clock axis. Obesity induced by the CAF is related to a chronic redox imbalance, and GSPE can reduce oxidative stress induced by the CAF by retraining the circadian rhythm of AOX enzyme activities disrupted by the diet. Also, GSPE was able to act as a chronotherapeutic agent and ameliorate pathological oxidative stress metabolic diseases.

Our last contribution was a study by Dave et al. investigating the effect of grapes entitled “Multi-Organ Nutrigenomic Effects of Dietary Grapes in a Mouse Model” (Contribution 13). In this original research article, the authors found that 12-month dietary grape feeding in mice equivalent to human consumption of 2.5 servings per day can alter the expression of over 35,000 genes throughout various organs including the kidney, colon, ovary and liver. Grape consumption leads to metabolic detoxification and ROS regulation in the liver, cellular metabolism and anti-inflammation in the ovary and kidney, and decreased mitochondrial dysfunction along with homeostatic balance in the colon. This study shows that grapes, due to their pleiotropic activities, can be viewed as a dietary agent that exemplify the power of dietary nutrigenomics.

In conclusion, the articles in this Special Issue emphasize the antioxidant properties of nature-derived materials and their potential role as treatment options for obesity and related diseases. The thirteen papers published describe various aspects of natural antioxidants. The original research articles provide important new features and review articles address recent advances in this area. We would like to express our gratitude to all of the authors and reviewers who had contributed to this Special Issue. We also thank the *Antioxidants* journal for giving us the opportunity to launch a Special Issue. We sincerely hope this Special Issue has helped readers to better understand the role of natural antioxidants in obesity and related diseases.
